# Influence of Mix Composition on the Microstructural Evolution of Leached Cement Pastes

**DOI:** 10.3390/ma19122664

**Published:** 2026-06-21

**Authors:** Kailai Zhang, Wenwei Li, Huamei Yang, Dan Tian, Jinyang Cui, Hao Wang, Fan Li

**Affiliations:** Hubei Key Laboratory of Water Engineering Materials and Application Technology, China Three Gorges Corporation, No.1, Liuhe Road, Wuhan 430010, China; zhang_kailai@ctg.com.cn (K.Z.);

**Keywords:** leaching, cement paste, fly ash, silica fume, pore structure, diffusion coefficient

## Abstract

Calcium leaching increases the hydraulic concrete material’s porosity and the diffusion coefficient, thereby jeopardizing engineering safety. Fly ash and silica fume are commonly used mineral admixtures in hydraulic concrete, and their effects on the material’s leaching characteristics, especially its microstructural and transport properties, require further investigation. In this study, calcium leaching tests were conducted on cement paste (CP), silica fume–cement paste (SF), and fly ash–cement paste (FA) using a 6 mol/L ammonium chloride solution to accelerate the leaching process. Subsequently, a series of quantitative and qualitative analyses was performed on the deteriorated specimens, including phenolphthalein indicator spraying, X-ray diffraction (XRD), nuclear magnetic resonance (NMR), and scanning electron microscopy (SEM). Additionally, the diffusion coefficients of the material at different locations were calculated and analyzed. The results show that partially replacing cement with silica fume or fly ash increases the initial porosity, gel pore content, and initial diffusion coefficients. After 28 days of leaching, compared to the initial values, the porosity increases in the 0–4 mm layer from the leached surface were 83.6% for CP, 11.0% for SF, and 39.0% for FA. The diffusion coefficients increased by factors of 14.3 (CP), 6.1 (SF), and 13.6 (FA), indicating enhanced resistance to leaching. The primary reason for this is that the reactive silica in the admixtures undergoes a pozzolanic reaction with the calcium hydroxide generated by cement hydration, producing additional calcium silicate hydrate (C-S-H) gel, which reduces the capillary pores that would otherwise result from calcium hydroxide decomposition.

## 1. Introduction

The pH of the pore solution in hydraulic concrete is about 12.5, while environmental water is often considered neutral, with a pH = 7. During the long-term service, in a low-alkali environment, cementitious substances in concrete, such as calcium hydroxide and calcium silicate hydrate, can decompose and precipitate, a phenomenon known as calcium leaching. Calcium leaching can increase porosity and the diffusion coefficient and decrease strength, seriously affecting engineering safety and efficiency. Leaching is an unavoidable issue for hydraulic concrete during the long-term operation. As long as the concrete is in contact with environmental water, leaching will occur, leading to deterioration. In China, many old dams that have been in service for many years are gradually showing signs of erosion and deterioration, e.g., the Fengman concrete gravity dam [1], Luowan concrete gravity dam [2], Shimantan roller-compacted concrete gravity dam [3], and Gutianxi Grade III flat-slab buttress dam [4].

Calcium leaching is a complex process involving multiple chemical reactions of hydration products. Both internal and external factors influence the leaching deterioration of cement-based materials. Numerous researchers have employed macroscopic and microscopic experimental techniques, such as X-ray diffraction (XRD), scanning electron microscopy (SEM), mercury intrusion porosimetry (MIP), and the Brunauer–Emmett–Teller (BET) specific surface area test, to investigate the effects of internal factors (e.g., cement type and mineral admixtures [5,6], water-to-cement ratio [7], sand content [8,9], compactness and pore characteristics of concrete specimens [10,11,12,13,14,15], and cracking conditions [16,17]), as well as external factors (e.g., solution type [18,19], temperature [20,21,22,23], water pressure and hydraulic gradient [24,25], and stress state [26,27]), on the leaching process. Among these factors, the water-to-cement ratio of the cement-based material and water quality have the greatest influence on the leaching process.

Existing research on calcium leaching tests has focused primarily on leaching depth and the degradation of mechanical properties. Only in a limited number of studies were the evolution of transportation characteristics resulting from changes in pore structure and the composition of cementitious materials during leaching investigated. Kamali et al. [8] conducted leaching tests on cement paste specimens under pure water conditions, considering different cement types, water-to-cement ratios, and temperatures, and proposed a one-dimensional model for leaching depth as a function of leaching time, incorporating the effects of cement type, water-to-cement ratio, solution type, and temperature. Heukamp et al. [27] performed triaxial tests on leached cement paste specimens, showing that the mechanical behavior of the degraded cement paste becomes highly sensitive to pore-water pressure. Wan et al. [28,29] established a solid–liquid equilibrium equation based on accelerated contact leaching tests of cement paste in ammonium nitrate solution. Kong et al. [30] studied the leaching process in the interlayer of roller-compacted concrete, and established a shear strength decay model. Phung et al. [31] conducted calcium leaching tests on cement paste with different limestone powder contents under accelerated conditions, and investigated the evolution of the material’s microstructural and transport properties during leaching by means of mercury intrusion porosimetry (MIP), BET specific surface area measurement, X-ray diffraction (XRD), scanning electron microscopy (SEM), etc. The results indicate that the leaching of cementitious phases significantly alters the pore structure of the cement paste, increasing both the diffusion coefficient and the permeability coefficient.

Since the leaching tests in deionized water require a long time, physical experiments mostly employ accelerated leaching methods, such as the use of chemical reagents [32,33,34,35,36] or electro-accelerated testing [37,38]. The chemical reagent method primarily uses ammonium salts to accelerate calcium leaching, such as ammonium chloride [35,36] and ammonium nitrate [32,33,34]. Gonçalves et al. [37] first immersed cement pastes in ammonium nitrate solutions at concentrations ranging from 0 to 50% and reported that 0.5% and 5.0% are the most aggressive. Song et al. [38] used a 6 mol/L ammonium chloride solution to accelerate leaching. They studied the effect of leaching on the diffusion coefficient of cement paste, finding that it increases most rapidly in the leached front region. The electrochemical method accelerates leaching by applying a voltage to the specimen, which promotes the migration of calcium ions out of the material [39]. This method was first adopted by Faucon [40]. Saito et al. [41,42] used the electrochemical method to accelerate mortar leaching to analyze its permeability and compressive strength, and plotted relationships between the permeability coefficient, compressive stress, and porosity. Currently, the most widely used method worldwide for accelerating the leaching process is the use of ammonium salt solutions.

Fly ash and silica fume are common mineral admixtures in concrete and are widely used in hydraulic structures. The objective of this study was to investigate the effects of replacing cement with fly ash and silica fume on the calcium-leaching characteristics of the materials, particularly the mechanisms underlying the evolution of material pore structure and diffusivity. In addition, cement-based materials often exhibit strong spatiotemporal variability during leaching, so it is necessary to sample and test at different leaching depths to reflect true leaching conditions better. At present, there are few reports on related research in the literature, and the pore structure and transmission characteristics of materials are closely related to their structural safety and durability, which requires in-depth research [43,44,45,46].

Owing to the modification of leaching characteristics by the incorporation of fly ash and silica fume, in this study calcium-leaching tests were conducted on different types of cement pastes (plain cement paste, fly ash–cement paste, and silica fume–cement paste). The evolution of the material’s mineral composition and microstructure induced was systematically investigated. The experimental procedure includes the following. Firstly, an ammonium chloride-accelerated calcium-leaching test was conducted, followed by sampling and testing, including using the g phenolphthalein spray method to assess dissolution depth, using NMR for pore structure analysis, using XRD for phase analysis, and using SEM to study the apparent morphology. Finally, the diffusion coefficients of materials in different parts were calculated and analyzed.

## 2. Materials and Methods

### 2.1. Materials

The specimens prepared for this test consisted of three types of cement-based materials: plain cement paste (CP), fly ash–cement paste (FA), and silica fume–cement paste (SF). The cement used is Hailuo PO42.5 ordinary Portland cement, and the mixing water is deionized (pH = 7). The chemical composition of the cement is provided in Table 1. The silica content of the fly ash is 43.8%, while that of the silica fume is 94.2%.

The design principle for the mix proportions is to maintain a constant water-to-binder ratio of 0.5. By incorporating the fly ash and silica fume, we aim to facilitate a reaction between the calcium hydroxide (CH) produced during cement hydration and the SiO_2_ present in the admixtures. This approach allows for an evaluation of how replacing part of the cement with mineral admixtures affects the material’s leaching resistance. The chemical compositions of the fly ash and silica fume are detailed in Table 2 and Table 3, respectively. The specific mix proportions used in the test are shown in Table 4.

According to the Chinese code Technical specification of fly ash for use in hydraulic concrete (DL/T 5055-2024) [47], for structural concrete, the maximum replacement ratio of fly ash in ordinary Portland cement should not exceed 30% of the total mass of cementitious materials. According to the code Technical specification of silica fume for use in hydraulic concrete (DL/T 5777-2018) [48], the maximum replacement ratio of silica fume shall not exceed 10% of the total mass of cementitious materials. Accordingly, the replacement ratios of both mineral admixtures adopted in this paper are approximately 60% of their respective allowable maximum values. Admittedly, these two replacement ratios can only qualitatively analyze the influence of different mineral admixtures on material properties. To determine the optimal replacement ratios of fly ash and silica fume from the perspective of leaching durability, a larger set of experiments is required. Furthermore, the water-to-cement ratio of the paste was chosen as 0.5, which is based on the Specification in Test method of cement mortar strength (ISO method) (GB/T 17671-2021) [49], where the water-to-cement ratio of the specimen is 0.5; therefore, the same ratio was adopted in this study.

### 2.2. Methods

The leaching process of cement-based materials is driven by diffusion. The calcium compounds in the solid skeleton phases and calcium ions in the pore solution are in equilibrium. In other words, the contents of CH and C-S-H in the cement-based material matrix have a one-to-one correspondence with the calcium ions in the pore solution, and this relationship is described by the solid–liquid equilibrium equation. Compared to deionized water, the direct effect of using a 6 mol/L ammonium chloride solution on the leaching process is the alteration in the critical concentration in the solid–liquid equilibrium equation, which consequently changes the leaching rate. However, the decomposition products and the sequence of solid calcium phases in the cement-based material remain unchanged. This method was adopted by several researchers [35,36,37,38], and it was employed in the present study, as well.

In this study, 12 specimens were prepared for each type of cement paste. At each leaching time (7, 14, 21, and 28 days), three specimens were taken to measure the leaching depth, and the average of these three measured values was used as the leaching depth at that time point. For the SEM observation, at least three different locations on the leached specimens were examined, and representative images were captured. XRD and NMR analyses were performed once on the sampled locations.

The experimental process of this study is as follows: first, cement paste, fly ash–cement paste, and silica fume–cement paste specimens were prepared according to the mix proportions, and then cured in saturated calcium hydroxide solution for 28 days at 20 °C. After the maintenance was complete, the specimen was sealed to create a one-dimensional dissolution environment. Then, the contact dissolution tests were conducted and samples were collected for testing during dissolution. The sampling depth ranges were 0–4 mm, 6–10 mm, and 13–17 mm, respectively.

The leaching depth was measured using phenolphthalein alcohol solution. Phenolphthalein alcohol solution appears purple-red under alkaline conditions. The pore solution of the leached sample is neutral and will not appear purple-red. However, for incomplete leached samples, due to the presence of calcium hydroxide, the pore solution remains alkaline and turns red when sprayed with phenolphthalein alcohol solution.

The phase composition was analyzed using X-ray diffraction (XRD). The X-ray diffractometer used is the Ultima IV model produced by RIGAKU Corporation in Japan. In this study, the samples were scanned over the 2θ range of 5–70. The step size is 0.01°, and the scanning speed is 2°/min.

The pore structure was determined using a nuclear magnetic resonance instrument. In this experiment, the MesoMR12-040H-I nuclear magnetic resonance instrument was used, produced by Suzhou Neway Analytical Instrument Co., Ltd. (Suzhou, China). The echo time for this test is 0.20 ms, with 64 scans. The aperture distribution is obtained by inverting the transverse relaxation time T2 spectrum, with a T2 range of 0.01~10,000 ms.

**Figure 1:**
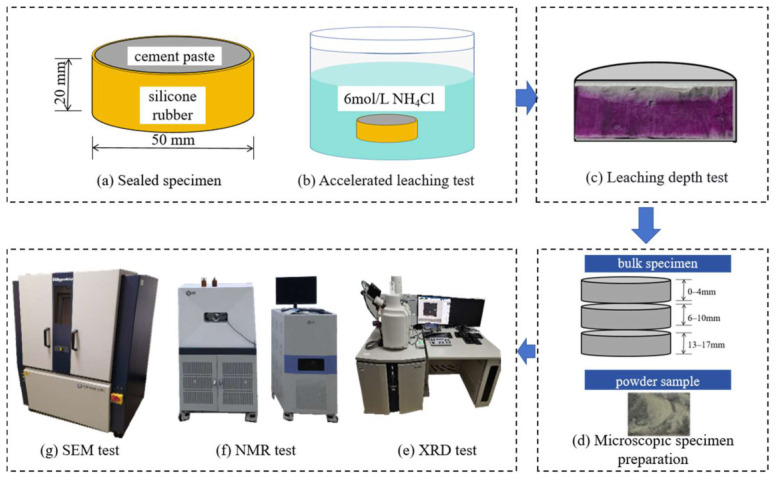
Experimental procedure.

The apparent morphology was observed using a scanning electron microscope. The equipment used is the SU3500 scanning electron microscope produced by Hitachi (Tokyo, Japan). The acceleration voltage used in this test is 5 kV, the working distance is 8 mm, and the magnification is 20,000 times. The experimental procedure is shown in Figure 1.

## 3. Experimental Result Analysis

### 3.1. Leaching Depth

**Figure 2:**
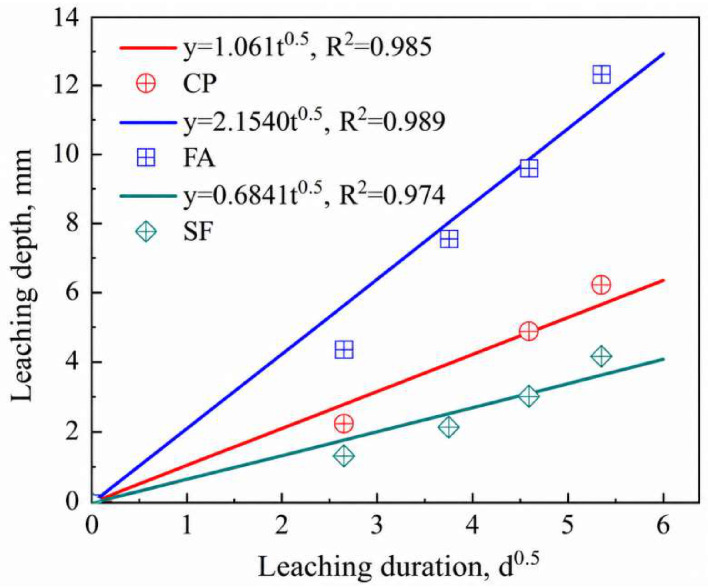
Degradation depth evolution with square root of time under accelerated leaching.

The leaching depth of cement-based materials is an important issue related to structural safety. When calcium hydroxide in cement-based materials is completely leached, the pore solution gradually loses its alkalinity, leading to the corrosion of reinforcing steel and cracking of concrete. Figure 2 presents the evolution of leaching depth and the corresponding fitting curves for the plain cement paste, fly ash–cement paste, and silica fume–cement paste under ammonium chloride accelerated-leaching conditions. The figure shows that the leaching depth is proportional to the square root of time, consistent with results reported in the literature [31]. The leaching depth of fly ash–cement paste develops the fastest, followed by plain cement paste and silica fume–cement paste. After 28 days of leaching, the leaching depths of cement paste, silica fume–cement paste, and fly ash–cement paste were 6.26 mm, 4.24 mm, and 12.6 mm, respectively.

From the perspective of leaching depth, it is evident that the cement paste containing silica fume exhibits better leaching resistance than both fly ash–cement paste and plain cement paste. This is because silica fume contains a large amount of reactive silica dioxide, which reacts with calcium hydroxide generated during cement hydration. This reaction reduces the amount of calcium hydroxide available for leaching, converting it into calcium silicate hydrate, which is more resistant to leaching, thereby improving the material’s leaching resistance. Fly ash–cement paste also helps to reduce the amount of calcium hydroxide produced during hydration. However, since the original mix proportion maintained a constant water-to-binder ratio, the water-to-cement ratio increased. As a result, the leaching resistance of fly ash–cement paste is lower than that of plain cement paste.

### 3.2. Content of Cementitious Materials

**Figure 3:**
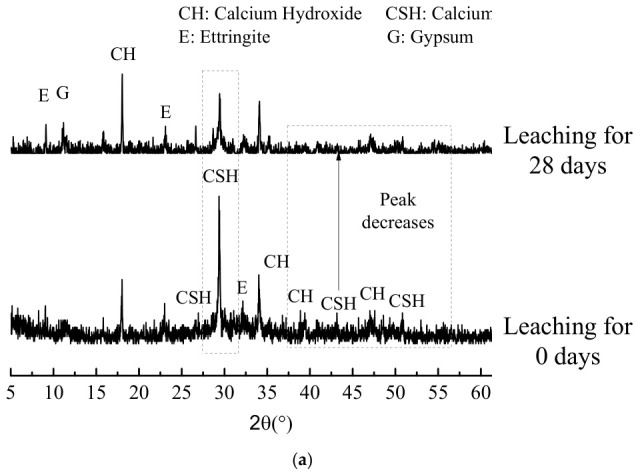

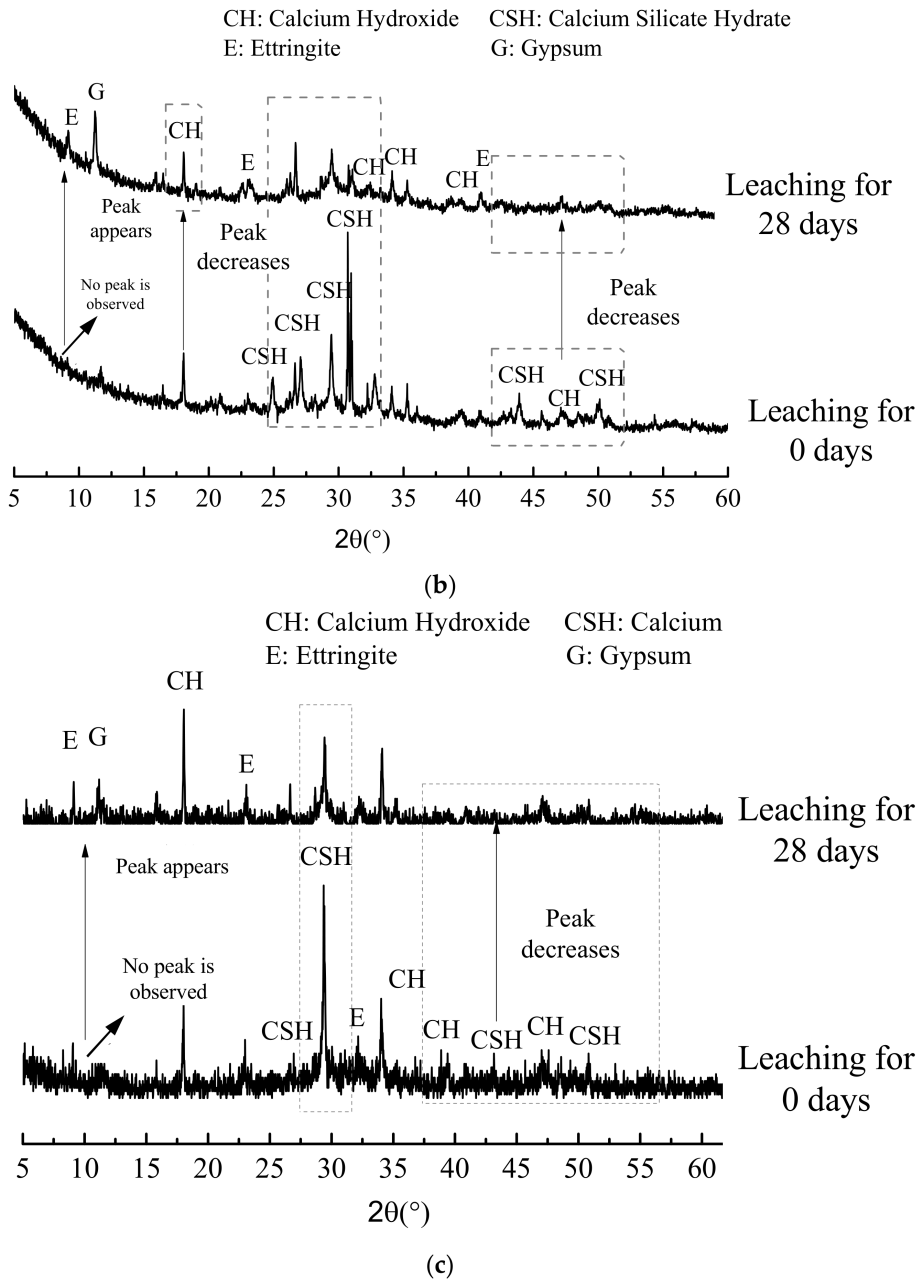
XRD result comparison of intact and leached samples: (**a**) cement paste; (**b**) silica fume–cement paste; (**c**) fly ash–cement paste.

Figure 3a presents the XRD patterns of cement paste specimens before and after 28 days of leaching within the diffraction angle range of 5°~70°. By comparing the changes in hydration products before and after leaching, the leaching process can be revealed. Before leaching, the specimens contained a large amount of C-S-H gel, CH, and Aft. The main peak in C-S-H gel was located at 28°~30°, with distinct peaks also present between 42°~52°; the main peak in CH was at 18° and 34°; and the main peak in Aft was at 34°. After 28 days of leaching, the most significant change was the reduction in the height of the C-S-H main peak near 28°~30°, along with a decrease in the peak at 42°~52°, indicating partial dissolution of the C-S-H gel. For CH, the peak near 18° showed no significant change, whereas those at 34°, 39°, and 47° decreased markedly. The peak of Aft near 32° nearly disappeared. These results indicate that under the leaching conditions, C-S-H gel, CH, and Aft underwent varying degrees of dissolution.

Figure 3b presents the XRD patterns of silica fume–cement paste within the diffraction angle range of 5° to 60° before and after leaching. Compared to pure cement paste, the silica fume–cement paste exhibits more abundant C-S-H gel peaks: distinct hump peaks are observed at 25°~35° and 42°~50°, indicating a higher C-S-H gel content. In contrast, CH only shows a relatively prominent peak near 18°. After 28 days of leaching, the hump peaks of the C-S-H gel in the 25°~35° and 42°~50° regions decrease significantly, and the main CH peak at 18° also shows a certain degree of reduction.

Figure 3c presents the XRD patterns of fly ash–cement paste before and after dissolution within the diffraction angle range of 5° to 70°. The peaks in C-S-H gel in fly ash–cement paste are fewer than those in silica fume–cement paste and primarily distributed at around 28° to 30°, with some broadened peaks near 43° and 51°. The peaks in CH are mainly located around 18° and 34°, while the peaks in ettringite are predominantly near 32°. After 28 days of calcium leaching, the main and broadened C-S-H gel peaks were significantly reduced, similar to the previous two types of specimens. The peaks in Aft were almost eliminated, while the peak in CH at 34° slightly decreased, with little change in the peak at 18°. The insignificant variation in the latter may be related to errors during the overall sampling of the specimens.

### 3.3. Evolution of the Pore Structure

Pores in cement-based materials can be classified according to pore size into gel pores (<10 nm), small capillary pores (10–50 nm), large capillary pores (50–100 nm), and air voids (>100 nm). Among these, capillary pores significantly influence the material’s permeability, while gel pores and air voids primarily affect its mechanical properties.

**Figure 4:**
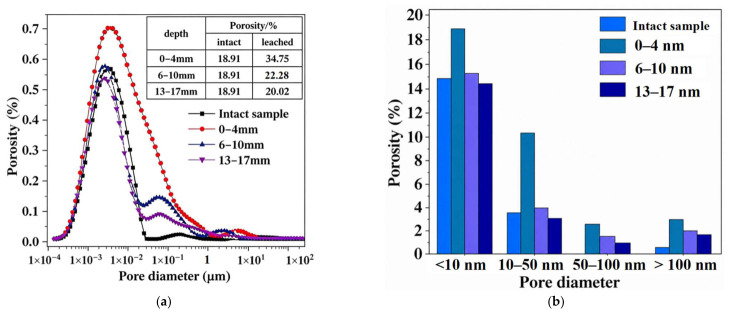
Evolution of cement paste pore structures at different positions after 28 days of leaching: (**a**) pore size distribution; (**b**) percentage of different pores.

To understand the changes in the microstructure of cement paste, nuclear magnetic resonance (NMR) tests were performed on different regions of cement paste specimens after 28 days of leaching. The sampled regions were 0–4 mm, 6–10 mm, and 13–17 mm, designated as the upper, middle, and lower parts, respectively. The changes in pore structure in different regions of the cement paste are compared in Figure 4. The figure shows that after 28 days of leaching, the porosity in the upper parts of the cement paste increased from 18.91% to 34.75%. The pore size distribution curve shifted significantly to the right, and the content of large pores increased notably. In the middle and lower parts, porosity increased from 18.91% to 22.28% and 20.02%, respectively. The pore size distributions in these regions were generally consistent with those of the unleached cement paste, with an increase in pore content in the 0.03–0.1 μm range. Based on the pore classification method described above, the pore size distributions in different regions of the cement paste after 28 days of leaching are summarized in Figure 4b. The figure shows that in the upper parts, the contents of all pore types increased significantly, indicating that both CH and C-S-H gel leached at the surface of the cement paste. In the middle and lower parts, the gel pore content remained essentially unchanged, and only a small amount of CH leaching led to an increase in the capillary pore and air void contents in the middle and lower parts.

**Figure 5:**
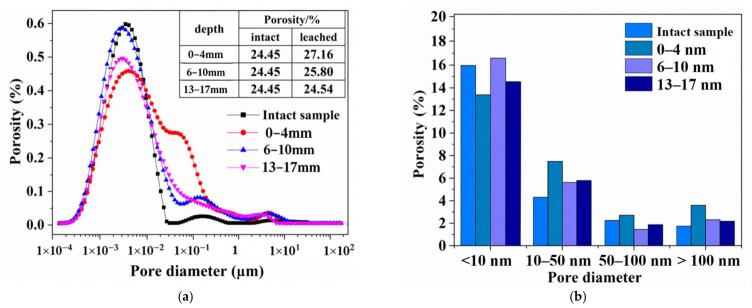
Evolution of silica fume–cement paste pore structures at different positions after 28 days of leaching: (**a**) pore size distribution; (**b**) percentage of different pores.

Figure 5 shows the changes in pore structure in different regions of silica fume–cement paste after 28 days of leaching. From the pore-size distribution curves, the material’s initial porosity is 24.45%. After 28 days of leaching, the porosity of upper, middle, and lower parts increases to 27.16%, 25.80%, and 24.54%, respectively. The pore-size distribution curves shift gradually to the right with increasing leaching time. In the upper part (0–4 mm) of the silica fume–cement paste, small capillary pores in the 0.01–0.1 μm range increase significantly, indicating that the permeability coefficient will increase markedly. The middle and lower parts of the silica fume–cement paste do not show this characteristic, suggesting that they have not been significantly leached. From the pore type proportion diagram, it can be seen that the proportion of gel pores in the upper part is smaller than that in the unleached specimen, indicating partial leaching of calcium silicate hydrate. The proportions of small capillary pores, large capillary pores, and macropores are all higher than those in other specimens, indicating that the transport characteristics of the upper part of the silica fume–cement paste have significantly increased. The middle part has the highest proportion of gel pores, possibly because some unhydrated cement continues to hydrate, producing more gel pores, or because the silicon dioxide in the silica fume reacts with CH to form new calcium silicate hydrate, thereby increasing the number of gel pores. The proportions of small capillary pores, large capillary pores, and air voids in the middle and lower parts of the silica fume–cement paste are relatively similar to each other and slightly larger than those of the unleached specimen, indicating that leaching is not evident in these regions. Compared to the plain cement paste, the pore size in the upper part of the silica fume–cement paste is larger than that in the upper part of the plain cement paste. Replacing cement with silica fume, while keeping the water-to-binder ratio constant and increasing the water-to-cement ratio, can enhance the paste’s erosion resistance.

**Figure 6:**
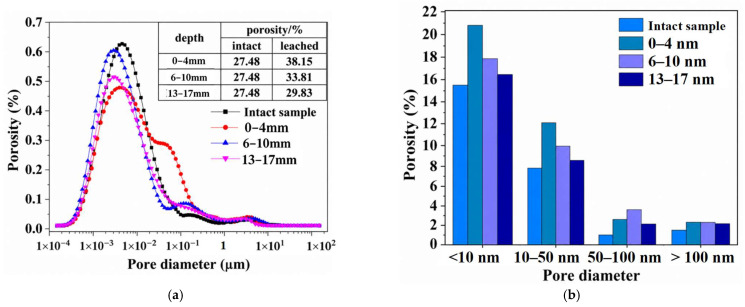
Evolution of fly ash–cement paste pore structures at different positions after 28 days of leaching: (**a**) pore size distribution; (**b**) percentage of different pores.

Figure 6 shows the changes in pore structure in different regions of fly ash–cement paste after 28 days of leaching. From the pore-size distribution curves, the material’s initial porosity is 27.48%. After 28 days of leaching, the porosity of the upper, middle, and lower parts increases to 38.15%, 33.81%, and 29.83%, respectively. The pore-size distribution curves shift gradually to the right with increasing leaching time. In the upper part of the fly ash–cement paste, small capillary pores around 0.1 μm increase significantly, indicating that its transport characteristic will increase markedly. The middle and lower parts of the fly ash–cement paste do not exhibit this characteristic, suggesting they have not been significantly leached. From the pore type proportion diagram, it can be seen that in the upper part, the proportions of gel pores and small capillary pores are smaller than those in the unleached specimen. In comparison, the proportions of large capillary pores and air voids are larger than those in the unleached specimen. The middle part has the highest proportion of gel pores, lower proportions of small and large capillary pores, and a proportion of air voids that is smaller than that of the upper part but larger than that of the unleached specimen. The pore type proportions in the lower part are relatively close to those of the unleached specimen, indicating that the lower part has not been leached. Compared to the plain cement paste, the pore size in the upper part of the fly ash–cement paste is larger than that in the upper part of the plain cement paste. Replacing cement with fly ash while keeping the water-to-binder ratio constant, but increasing the water-to-cement ratio, does not enhance the paste’s erosion resistance.

As indicated above, after incorporating the silica fume and fly ash, the initial porosity of the material increased from 0.189 to 0.244 and 0.274, corresponding to increases of 29% and 45%, respectively. The porosity increment per unit mass of cement replaced was 4.4% for silica fume and 2.0% for fly ash. After 28 days of leaching, in the 0–4 mm region from the leached surface, porosity increased to 0.347 (CP), 0.271 (SF), and 0.381 (FA). Compared with the initial porosity of the plain cement paste, the porosity increases in the specimens were 83.6% (CP), 43.3% (SF), and 101% (FA); relative to their own initial porosity values, the increases were 83.6% (CP), 11.0% (SF), and 39.0% (FA). Therefore, although replacing a portion of cement with silica fume and fly ash increases the material’s initial porosity, it effectively mitigates porosity growth during leaching.

### 3.4. Surface Morphology

**Figure 7:**
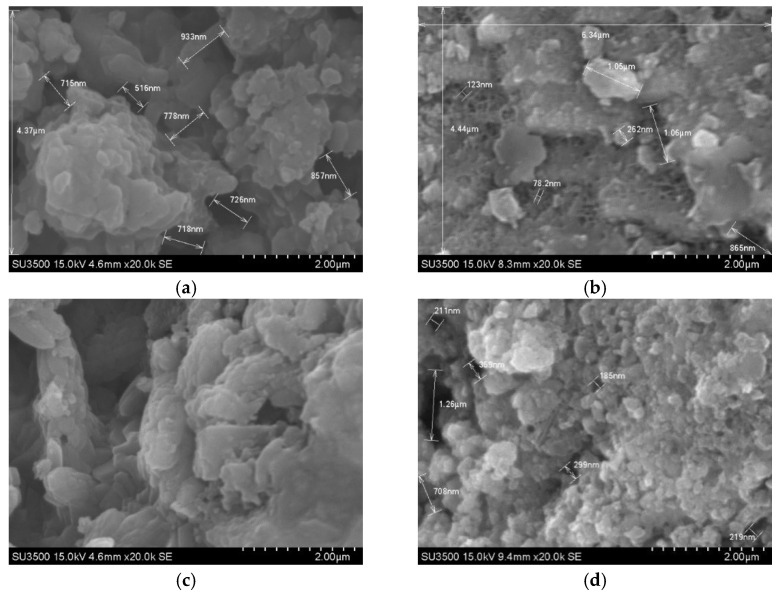
Scanning electron microscope of cement paste surface: (**a**) intact plain cement paste sample, (**b**) leached plain cement paste sample, (**c**) intact silica fume–cement paste sample, and (**d**) leached silica fume–cement paste sample.

Figure 7 shows scanning electron microscopy (SEM) images of the surface of the unleached cement paste specimen and the specimen after 28 days of leaching, at a magnification of 20,000 times. Figure 7a shows that the surface of the unleached plain cement paste specimen contains abundant, thick, and large CH deposits, with pore diameters mostly ranging from 500 nm to 1 μm. After 28 days of leaching, the typical CH crystals were destroyed and leached into a porous, flaky form, exposing cement particles (Figure 7b). Figure 7c presents SEM images of the surface of the unleached silica fume–cement paste specimen and the specimen after 28 days of leaching. It can be observed that the surface of the unleached specimen has fewer CH deposits. After 28 days of leaching, the irregular “large particle” crystals on the paste surface dissolved into loose small particles, a morphology completely different from that of leached CH in plain cement paste (Figure 7d). This is because the silica fume particles in the paste do not participate in leaching and exhibit only a slight surface “leaching” effect.

During the leaching, the diffusion coefficient increases continuously with increasing porosity. The effective diffusion coefficient model was first proposed by Garboczi and Bentz [50], as shown in the following:

(1) $$D=\left[0.001+0.07\phi_{\mathrm{cap}}^{2}+1.8H\left(\phi_{\mathrm{cap}}-\phi_{c}\right)\left(\phi_{\mathrm{cap}}-\phi_{c}^{2}\right)\right]D_{0},$$

where *H*(*x*) denotes the weighting function, where *H*(*x*) = 1 for *x* > 0 and *H*(*x*) = 0 for *x* < 0, and *x* is the argument of the function; $\phi_{\mathrm{cap}}$ is the capillary porosity, $\phi_{c}$ is the critical porosity (set to 0.18); and $D_{0}$ is the diffusion coefficient of calcium ions in water (m^2^/s).

However, this model was primarily developed to calculate the evolution of the diffusion coefficient due to continued cement hydration and does not apply to cement-based materials after leaching, since the effect of leaching on porosity is much greater than that of late-age cement hydration. Gérard [51] proposed an evolution equation for the diffusion coefficient that accounts for different calcium compounds fractions and also incorporates the effect of continued cement hydration, as shown in the following:

(2) $$D=D_{0}\left(\frac{D_{S}}{D_{0}}\right)V^{\frac{\beta V_{\mathrm{CH}}^{d}+\alpha V^{d}}{V_{\mathrm{CH}}^{d}+V^{i}}},$$

where *D* is the current diffusion coefficient (m^2^/s); $D_{0}$ is the initial diffusion coefficient (m^2^/s); $D_{s}$ is the diffusion coefficient after complete leaching (m^2^/s); $V^{i}$ is the volume generated by hydration in the hydrated cement paste minus the volume occupied by CH and SiO_2_; $V_{CH}^{d}$ is the volume occupied by CH; $V_{d}$ is the volume occupied by C-S-H; and *α* and *β* are parameters related to cement hydration.

Van Eijk and Brouwers [52] proposed an improved relationship between the porosity and the effective diffusion coefficient based on previous work, as shown in the following:

(3) $$\begin{array}{c} \frac{D_{e}}{D_{0}}=0.0025-0.07\varphi_{\mathrm{cap}}^{2}-0.18H\left(\varphi_{\mathrm{cap}}-0.18\right){\left(\varphi_{\mathrm{cap}}-0.18\right)}^{2}\\ +0.14\varphi_{\mathrm{cap}}^{2}+3.6H\left(\varphi_{\mathrm{ca}p}-0.16\right){\left(\varphi_{\mathrm{cap}}-0.16\right)}^{2} \end{array},$$

where $D_{e}$ is the effective diffusion coefficient of calcium ions (m^2^/s); $D_{0}$ is the diffusion coefficient of calcium ions in water (m^2^/s); and $\varphi_{\mathrm{cap}}$ is the capillary porosity.

**Figure 8:**
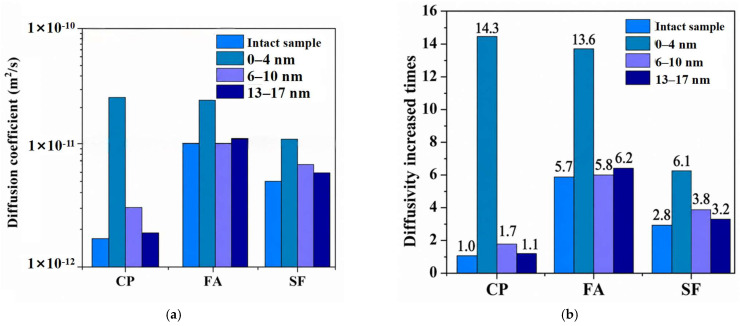
Evolution of diffusion coefficient in different zones of hardened cement paste before and after 28-day leaching: (**a**) diffusion coefficient in different zones; (**b**) increase factor of diffusion coefficient in different zones.

Based on the porosity of the cement paste after leaching, the evolution of the diffusion coefficient of different cement paste before and after 28 days of leaching is shown in Figure 8a using Equation (3). The figure shows that at the initial stage, the diffusion coefficients for plain cement paste, fly ash–cement paste, and silica fume–cement paste are 1.73 × 10^−12^ m^2^/s, 9.86 × 10^−12^ m^2^/s, and 4.9 × 10^−12^ m^2^/s, respectively. Obviously, under the constant water-to-binder ratio, replacing cement with fly ash or silica fume increases the initial diffusion coefficient of the specimens. After 28 days of leaching, for the specimens at 0–4 mm, the leaching-depth evolution results indicate that calcium hydroxide in this range has essentially been completely leached. The diffusion coefficients of plain cement paste, fly ash–cement paste, and silica fume–cement paste are 2.47 × 10^−11^ m^2^/s, 3.53 × 10^−11^ m^2^/s, and 1.05 × 10^−11^ m^2^/s, respectively. The diffusion coefficients of plain cement paste and fly ash–cement paste are very close to each other, while that of silica fume–cement paste is only about half that of the plain cement paste. For the specimens at 6–10 mm and 13–17 mm, since the degree of leaching is not severe, the diffusion coefficients still follow the same trend as the unleached specimens, with those of fly ash–cement paste and silica fume–cement paste being larger than that of plain cement paste.

Figure 8b shows increased times for the diffusion coefficient across different regions of the cement paste after 28 days of leaching, with the baseline being the cement paste’s initial diffusion coefficient. The figure shows that for specimens at 0–4 mm, the diffusion coefficient of plain cement paste increased by a factor of 14.3, while those of fly ash–cement paste and silica fume–cement paste increased by factors of 13.6 and 6.1, respectively. The reason for this significant difference is, on the one hand, that the initial diffusion coefficient of plain cement paste is relatively small. In contrast, those of fly ash– and silica fume–cement pastes are relatively large, leading to different increase factors under the same leaching effect. On the other hand, the addition of silica fume and fly ash consumes calcium hydroxide in the specimens. The leaching of calcium hydroxide generates capillary pores, which directly affect the diffusion coefficient. For the specimens at 6–10 mm, the increase factors for the plain cement paste, fly ash–cement paste, and silica fume–cement paste are 1.7, 5.8, and 3.8, respectively. For the specimens at 13–17 mm, the increase factors are 1.1, 6.2, and 3.2, respectively. From the evolution of the diffusion coefficient in these two regions, it can be seen that in regions with lower leaching, the diffusion coefficients of fly ash–cement paste and silica fume–cement paste are larger than those of plain cement paste, which is due to the change in water-to-cement ratio.

In summary, the difference in the increase factor of the diffusion coefficient is related to the degree of leaching. Under the condition of a constant water-to-binder ratio, a reduction in cement content leads to an increase in the initial diffusion coefficient. However, because the silica fume and fly ash react with the calcium hydroxide generated by cement hydration to form new calcium silicate hydrate, and the leaching decomposition of calcium silicate hydrate is obviously much more difficult than that of calcium hydroxide, this results in a lower increase factor for the diffusion coefficient under leaching. Therefore, provided that the initial diffusion coefficient meets the requirements, the appropriate incorporation of fly ash and silica fume can improve the leaching resistance of cement paste.

## 4. Mechanism Analysis

In this section, the effects of two admixtures, silica fume and fly ash, on the leaching of cement paste specimens were investigated. When silica fume is added to cement, a pozzolanic reaction occurs. The main reaction equation is [53]

(4) $$2S+3\mathrm{CH}\to C_{3}S_{2}H_{3},$$

where S is the SiO_2_; CH is the Ca(OH)_2_; and C_3_S_2_H_3_ is the C-S-H gel.

From Equation (4), n mol of SiO_2_ reacts with 1.5 n mol of Ca(OH)_2_ to produce 1 mol of C-S-H. In ordinary silica fume, the SiO_2_ content generally exceeds 90%, and the silica fume used in this study has a SiO_2_ content of 94.2%. Adding a specific amount of silica fume to the cement paste effectively reduces the Ca(OH)_2_ content in the cement hydration products. As the Ca(OH)_2_ content decreases, the number of capillary pores generated during leaching is reduced, thereby slowing the increase in the material’s porosity and diffusion coefficient and, consequently, improving its leaching resistance. The results of this study confirm this conclusion.

Compared to the silica fume, the composition of fly ash is more complex. Fly ash contains not only reactive SiO_2_ but also Al_2_O_3_. During the pozzolanic reaction of fly ash, the products include not only C-S-H but also AFt and C_4_AH_13_. The results of this study show that adding fly ash can improve the material’s leaching resistance to a certain extent. The main reaction equations for the pozzolanic reaction of fly ash are as follows [54]:

(5) $$\left\{\begin{array}{c} 2S+3\mathrm{CH}\to C_{3}S_{2}H_{3}\\ A+C\bar{S}H_{2}+3\mathrm{CH}+7H\to C_{4}A\bar{S}H_{12}\\ A+4\mathrm{CH}+9H\to C_{4}{\mathrm{AH}}_{13} \end{array}\right.,$$

where A is the Al_2_O_3_; H is the H_2_O; $C\bar{S}H_{2}$ is the CaO·SO_3_·H_2_O; $C_{4}A\bar{S}H_{12}$ is the 3CaO·Al_2_O_3_·CaSO_4_·12H_2_O; and C_4_AH_13_ is the 4CaO·Al_2_O_3_·12H_2_O.

The pozzolanic reactions of fly ash and silica fume exhibit significant differences in both rate and extent, which fundamentally account for their distinct leaching resistances. Specifically, the silica fume possesses an extremely high specific surface area (15–30 m^2^/g) and a high reactive SiO_2_ content (94.2% for the silica fume used in this study). As a result, it rapidly consumes a large amount of calcium hydroxide (CH) within hours to days, with fast reaction kinetics and a high degree of reaction. This leads to a significant refinement in the capillary pore structure at an early age, effectively inhibiting pore-coarsening induced by leaching. In contrast, fly ash typically has a much lower specific surface area (0.3–0.6 m^2^/g) and a lower reactive SiO_2_ content (43.8% for the fly ash used in this study). Moreover, its dense vitreous surface structure must first be attacked and dissolved by alkalis, resulting in a slow pozzolanic reaction (weeks to months) and a limited capacity for CH consumption at early ages. Consequently, its mitigating effect on porosity increases during leaching is restricted. In this study, the replacement ratios of the two supplementary cementitious materials were identical (both replacing a certain proportion of cement by mass). However, owing to its faster reaction and more complete CH consumption, the porosity increase in the 0–4 mm surface layer of the silica fume group was only 11.0%, whereas that of the fly ash group reached as high as 39.0%. Therefore, the most critical factor affecting the leaching resistance of these two materials is the specific surface area (particle size), followed by the reactive SiO_2_ content. Together, these two factors determine the pozzolanic reaction kinetics and the efficiency of CH consumption, ultimately leading to the substantial difference in leaching resistance.

Under the condition of a constant water-to-binder ratio, replacing a portion of cement with silica fume or fly ash increases the initial porosity and initial diffusion coefficient of the material, but simultaneously significantly alters the composition of the hydration products. Ordinary cement hydration produces a large amount of calcium hydroxide (CH), accounting for approximately 20–25% by volume of the hardened cement paste. In contrast, when the silica fume or fly ash is incorporated, the reactive silica contained therein undergoes a pozzolanic reaction with calcium hydroxide, generating additional calcium silicate hydrate (C-S-H) and consequently substantially reducing the calcium hydroxide content in the system.

During the leaching process, the material’s resistance to degradation is governed by two factors: (i) the ionic transport capacity (related to porosity and diffusion coefficient) and (ii) the decomposition rate of the calcium compounds in the solid skeleton. In ordinary cement paste, the abundant calcium hydroxide decomposes rapidly under leaching, forming interconnected capillary pores, accelerating calcium ion dissolution, and leading to a rapid increase in the leaching rate. In contrast, in specimens containing silica fume or fly ash, the calcium hydroxide content is significantly reduced, and the calcium ions released during leaching primarily derive from the slow decomposition of calcium silicate hydrate. Despite the higher initial porosity, the decomposition rate of C-S-H is much lower than that of CH; consequently, the overall leaching process is considerably slower, thus exhibiting superior long-term durability.

In conclusion, the initial porosity and diffusion coefficient are not the sole determinants of leaching durability. The type of hydration products and their inherent chemical stability play a more critical role in this process.

When using admixtures to enhance the leaching resistance of materials, silica fume can be used to partially replace cement, improving leaching resistance. If fly ash is used, cement should not be replaced; instead, it should be added as an extra portion while maintaining the original water-to-cement ratio to enhance the material’s leaching resistance.

## 5. Conclusions

In this paper, the effects of replacing cement with fly ash and silica fume on the calcium leaching characteristics of materials were investigated. Accelerated leaching tests (using ammonium chloride solution) were conducted on plain cement paste, fly ash–cement paste, and silica fume–cement paste. The evolution of leaching depth, chemical composition, pore structure, surface morphology, and diffusivity coefficient of the specimens was systematically analyzed. The main conclusions are as follows:

(1) The leaching depth is proportional to the square root of time. After 28 days of leaching, the leaching depths of cement paste, silica fume–cement paste, and fly ash–cement paste are 6.26 mm, 4.24 mm, and 12.6 mm, respectively.
(2) Partially replacing cement with silica fume or fly ash increases the initial porosity and gel pore content of the material, but slows down the rate of porosity increase during leaching. After 28 days of leaching, the porosity in the 0–4 mm layer from the leached surface increased to 0.347 (CP), 0.271 (SF), and 0.381 (FA). Compared with the initial porosity of the plain cement paste, the porosity increases in the specimens were 83.6% (CP), 43.3% (SF), and 101% (FA); relative to their own initial porosity values, the increases in porosity were 83.6% (CP), 11.0% (SF), and 39.0% (FA) compared to the initial values.
(3) The change in diffusion coefficient follows a similar pattern to that of porosity. Although the incorporation of silica fume or fly ash increases the initial diffusion coefficient, the degradation rate of specimens containing these mineral admixtures under leaching is lower than that of plain cement paste. After 28 days of leaching, the diffusion coefficients in the region 0–4 mm from the surface of plain cement paste, fly ash-blended cement paste, and silica fume-blended cement paste increase by factors of 14.3, 13.6, and 6.1, respectively, indicating enhanced resistance to leaching.
(4) The mechanism by which the fly ash and silica fume improve the leaching resistance of cement paste is as follows: the reactive silicon dioxide in the admixtures undergoes a secondary hydration reaction with calcium hydroxide generated from cement hydration, producing calcium silicate hydrate gel with a low calcium-to-silicon ratio. This process reduces the capillary pores formed by extensive leaching of calcium hydroxide, thereby suppressing the increase in the diffusion coefficient and delaying the leaching and migration of calcium ions.

Regarding the mix design of concrete structures serving in water environments, under the prerequisite of meeting basic requirements, such as strength and workability, it is recommended that engineers select fly ash or silica fume as mineral admixtures based on the strength grade required for the specific structural element. For mass concrete in dam bodies, the use of fly ash is recommended. This not only enhances the material’s resistance to leaching but reduces cement consumption, as well, thereby lowering hydration heat and avoiding thermal cracking. For special structural elements with abrasion resistance requirements, such as spillway tunnels and overflow dam sections, silica fume is recommended. Silica fume can improve both the leaching resistance and the strength and abrasion resistance of the material. It should be noted that the present study result does not provide a definitive optimal replacement ratio; in practice, the appropriate replacement ratio should be determined through further testing.

Although the aforementioned supplementary cementitious materials can significantly enhance the resistance of cement-based materials to leaching, they still face several major challenges in practical applications. The main challenges are that both materials increase sensitivity to moist-curing, and require design and regulation of the paste performance, as well as optimization of the mix proportion. Specifically, fly ash leads to slow early-age strength development and a prolonged setting time, and its quality is highly susceptible to fluctuations in the raw ash from power plants, with the pozzolanic reaction nearly ceasing at low temperatures. In contrast, due to its extremely high specific surface area, the silica fume drastically increases water demand, makes the paste more viscous and difficult to place, and significantly increases the risk of cracking caused by plastic and drying shrinkage.

## Figures and Tables

**Table 1:** Chemical composition of the cement (%).

Compounds	Content	Compounds	Content
CaO	67.58	SO_3_	2.63
SiO_2_	13.57	Cl^−^	0.021
Al_2_O_3_	5.05	Na_2_O	0.04
Fe_2_O_3_	3.64	Loss on ignition	0.37
MgO	1.79	Insoluble residue	2.39

**Table 2:** Chemical composition of the fly ash (%).

Compounds	Content	Compounds	Content	Compounds	Content
SiO_2_	43.80	K_2_O	2.28	P_2_O_5_	0.17
Al_2_O_3_	27.60	TiO_2_	1.58	BaO	0.10
CO_2_	14.70	MgO	0.61	MnO	0.04
Fe_2_O_3_	4.68	SO_3_	0.42	V_2_O_5_	0.04
CaO	3.52	Na_2_O	0.29	Cr_2_O_3_	0.04

**Table 3:** Chemical composition of the silicon fume (%).

Compounds	Content	Compounds	Content	Compounds	Content
SiO_2_	94.20	Al_2_O_3_	0.30	SO_3_	0.02
ZrO_2_	2.96	CaO	0.05	Na_2_O	0.02
CO2	1.46	TiO_2_	0.03	NiO	0.01
Fe_2_O_3_	0.56	K_2_O	0.03	Y_2_O_3_	0.01
P_2_O_5_	0.36	PbO	0.03		

**Table 4:** Mix proportion of slurry.

Slurry Type	Cement /kg	Mix Composition /kg	Powder/kg	Water /kg	Water Cement Ratio	Water Powder Ratio
CP	100.00	-	100.00	50.00	0.50	0.50
SF	93.43	6.57	100.00	50.00	0.54	0.50
FA	77.78	22.22	100.00	61.11	0.79	0.50

## Data Availability

The original contributions presented in this study are included in the article. Further inquiries can be directed to the corresponding author.

## Nomenclature

The following abbreviations are used in this manuscript:

CP	Cement Paste
SF	Silicon Fume–Cement Paste
FA	Fly Ash–Cement Paste
CH	Calcium Hydroxide
C-S-H	Calcium Silicate Hydrate gel
SEM	Scanning Electron Microscopy
NMR	Nuclear Magnetic Resonance
XRD	X-ray diffraction
S	SiO_2_
$C\bar{S}H_{2}$	CaO·SO_3_·H_2_O
$C_{4}A\bar{S}H_{12}$	3CaO·Al_2_O_3_·CaSO4·12H_2_O
C_4_AH_13_	4CaO·Al_2_O_3_·12H_2_O.
